# Exercise intolerance and impaired aerobic capacity in heart transplant recipients

**DOI:** 10.1113/EP093964

**Published:** 2026-10-09

**Authors:** Fred W. Kolkhorst, Daniel T. Cannon, Harry B. Rossiter

**Affiliations:** ^1^ School of Exercise and Nutritional Sciences San Diego State University San Diego California USA; ^2^ Respiratory Research Center The Lundquist Institute for Biomedical Innovation at Harbor−UCLA Medical Center Torrance California USA; ^3^ Division of Respiratory and Critical Care Medicine, Department of Medicine Harbor−UCLA Medical Center Torrance California USA

**Keywords:** calcineurin inhibitors, convective oxygen transport, heart denervation, muscle, oxygen diffusive capacity, ${\dot{V}}_{O_{2}\max}$

## Abstract

Heart transplant recipients exhibit reduced exercise tolerance and persistently diminished aerobic capacity despite exercise training. Exercise tolerance depends on integrated oxygen transport and utilization, which are impaired in heart transplant recipients. We synthesize evidence supporting that the combined effects of impaired pulmonary gas exchange, reduced haemoglobin concentration, heart denervation, chronic calcineurin inhibition, decreased muscle capillary density, and detraining from limited physical activity contribute to: (1) reduced pulmonary diffusion and alveolar ventilation–perfusion mismatch that contribute to hypoxemia and increased ventilatory demand; (2) impaired convective oxygen delivery due to anaemia and to heart denervation that limits cardiac output; (3) deficits in endothelial function and reduced muscle capillarization that contribute to diminished convective and diffusive oxygen transport within skeletal muscle. Using Wagner's model, which describes oxygen transport from the atmosphere to muscle mitochondrion, we develop the hypothesis that the persistent reduction in aerobic capacity in heart transplant recipients reflects the combined and interactive effects of impaired convective oxygen transport and reduced muscle oxygen diffusive capacity, rather than a single dominant limitation. This basis generates testable physiological targets for interventions aimed at improving functional capacity after transplantation.

## Abstract (highlights)

- **What is the topic of this review?**
  This review examines the oxygen cascade and evidence for or against impairment of aerobic capacity (${\dot{V}}_{O_{2}\mathrm{peak}}$) in heart transplant recipients (HTRs).
- **What advances does it highlight?**
  It is proposed that ${\dot{V}}_{O_{2}\mathrm{peak}}$ impairment in HTRs reflects the combined effects of impaired pulmonary gas exchange, heart denervation, chronic calcineurin inhibition and detraining from limited physical activity. The analysis justifies the need for direct, prospective measurement of these components in HTRs.

## 1 INTRODUCTION

Heart failure is a complex condition that results in a progressive structural and functional impairment of the heart (Jaski, 2015). When heart failure reaches an advanced stage (stage D, NYHA Class IV), heart transplantation is the definitive treatment for a select group of patients, primarily those with heart failure with reduced ejection fraction (HFrEF).

Following transplantation, there are documented reports of heart transplant recipients (HTRs) performing impressive physical feats including completing marathons (Haykowsky et al., 2014), Ironman triathlons (Haykowsky et al., 2018) and other extreme endurance events (Haykowsky et al., 2018; Niset et al., 1985). However, these reports represent outliers. The great majority of HTRs have an impaired aerobic capacity (${\dot{V}}_{O_{2}\max}$), even in individuals who were formerly athletic. At 1 year post‐transplant, ${\dot{V}}_{O_{2}\max}$ averages 16−20 mL kg^−1^ min^−1^ (Givertz et al., 1997; Habedank et al., 2007; Leung et al., 2003; Mandak et al., 1995), which is 40–60% below age‐matched healthy control individuals (Kao et al., 1994; Mandak et al., 1995; Scott et al., 2009), and ${\dot{V}}_{O_{2}\max}$ remains severely depressed at least 6 years following transplantation (Kao et al., 1995; Mandak et al., 1995; Tucker et al., 2018). A low ${\dot{V}}_{O_{2}\max}$ in HTRs may restrict activities of daily living (Goulding et al., 2021), limit exercise tolerance and is associated with increased risk of mortality (Hanff et al., 2023). Although exercise training in HTRs can increase ${\dot{V}}_{O_{2}\max}$, the training adaptations remain below those expected for age‐matched individuals. In a review of eight randomized controlled trials, peak oxygen uptake (${\dot{V}}_{O_{2}\mathrm{peak}}$) in HTRs was 19 mL kg^−1^ min^−1^ before training and increased by 2.8 mL kg^−1^ min^−1^ after moderate‐intensity aerobic training and 4.3 mL kg^−1^ min^−1^ with high‐intensity interval training (Foulkes et al., 2023). Most of these training programmes were of relatively short duration (8–26 weeks) so any increase of ${\dot{V}}_{O_{2}\max}$ would be expected to be modest. But, even after 1 year of exercise training (114 ± 18 training sessions), ${\dot{V}}_{O_{2}\mathrm{peak}}$ in 20 HTRs increased from 18.8 ± 4.2 to just 20.1 ± 4.2 mL kg^−1^ min^−1^ (*P <* 0.05) (Tegtbur et al., 2003). While the magnitude of these improvements is similar to those of older sedentary, healthy individuals (Huang et al., 2005), the absolute ${\dot{V}}_{O_{2}\mathrm{peak}}$ of HTRs still remains considerably below normal after training. These reports raise a critical question: what mechanisms limit aerobic capacity in HTRs despite exercise training?

Exercise tolerance depends largely on the capacity for oxygen transport and utilization (Wasserman et al., 2012; Houstis et al., 2018). Thus, efforts to identify limitations have focused on the transport of oxygen to active skeletal muscle and its utilization by skeletal muscle mitochondria. The pathway of oxygen transport can be described by four key steps in series: (1) alveolar ventilation and perfusion; (2) diffusion from the alveoli and loading onto haemoglobin; (3) cardiac output and muscle perfusion; and (4) diffusion from muscle capillaries to mitochondria (Wagner, 2023). Each component is critical in the oxygen cascade (Wagner, 1996b) and can contribute to limiting ${\dot{V}}_{O_{2}\max}$ (Wagner, 1996a). As these four steps and mitochondrial respiration are interdependent, it is challenging to identify the mechanism(s) that mediate limitations to ${\dot{V}}_{O_{2}\max}$. We propose that persistent exercise intolerance after heart transplantation reflects the combined effects of impaired pulmonary gas exchange, reduced haemoglobin concentration, heart denervation, chronic calcineurin inhibitor (CNI) therapy, decreased muscle capillary density, and detraining from limited physical activity, which together constrain both convective oxygen delivery and skeletal muscle oxygen diffusing capacity. Accordingly, we sought to synthesize current evidence within Wagner's convective–diffusive model to identify key physiological mechanisms that warrant direct experimental investigation.

## 2 ${\dot{V}}_{O_{2}\max}$ IS DETERMINED BY INTERACTIONS OF CONVECTIVE AND DIFFUSIVE OXYGEN TRANSPORT

Rather than being governed by a single limiting factor, the variables that mediate ${\dot{V}}_{O_{2}\max}$ are distributed throughout the convective and diffusive components of oxygen transport and mitochondrial respiration. Wagner's convective–diffusive model provides a comprehensive and quantitative approach that accounts for the delivery of oxygen via the circulation (convective oxygen transport) and its transfer into muscle tissue (diffusive oxygen transport) (Wagner, 1996a, 2023). Convective oxygen transport can be described by the Fick principle:

(1) $${\dot{V}}_{O_{2}}=\dot{Q}\times \left(C_{aO_{2}}-C_{vO_{2}}\right)$$

where ${\dot{V}}_{O_{2}}$ is the rate of oxygen uptake, $\dot{Q}$ is cardiac output, $C_{aO_{2}}$ is arterial oxygen concentration, and $C_{vO_{2}}$ is muscle venous oxygen concentration. Oxygen delivery to the tissues is therefore dependent on both blood flow and oxygen concentration in arterial blood. Muscle diffusion, representing oxygen transport from the capillaries to the mitochondria, is described by Fick's law of diffusion:

(2) $${\dot{V}}_{O_{2}}=D_{mO_{2}}\times \left({\bar{P}}_{\mathrm{cap}O_{2}}-P_{\mathrm{mit}O_{2}}\right)$$

where $D_{mO_{2}}$ is the diffusing capacity of muscle for oxygen, ${\bar{P}}_{\mathrm{cap}O_{2}}$ is mean capillary partial oxygen pressure and $P_{\mathrm{mit}O_{2}}$ is mitochondrial partial oxygen pressure. At metabolic rates near ${\dot{V}}_{O_{2}\max}$, $P_{\mathrm{mit}O_{2}}$ is presumed to approach zero and can be eliminated (Wagner, 2023). Assuming ${\bar{P}}_{\mathrm{cap}O_{2}}$ is proportional to muscle venous partial oxygen pressure ($P_{\mathrm{mv}O_{2}}$), a commonly used approximation in the Wagner model, then Equation 2 simplifies to:

(3) $${\dot{V}}_{O_{2}}=D_{mO_{2}}\times P_{\mathrm{mv}O_{2}}\times k$$

where *k*, a proportionality constant relating mean muscle partial oxygen pressure to $P_{\mathrm{mv}O_{2}}$, is ∼2 (Wagner, 2008).

Wagner's model defines ${\dot{V}}_{O_{2}\max}$ at the intersection of the convective and muscle diffusive components plotted as a function of $P_{\mathrm{mv}O_{2}}$ (Figure 1). The convective relationship reflects systemic oxygen transport that is influenced predominantly by the oxygen−haemoglobin dissociation relationship and $\dot{Q}$; the muscle diffusive relationship is a linear function intercepting the origin, given the assumption that $P_{\mathrm{mit}O_{2}}$ approximates 0 mmHg. At ${\dot{V}}_{O_{2}\max}$ (or for any given ${\dot{V}}_{O_{2}}$), mass balance demands that the solutions to Equations 2 and 3 are equal, and therefore the point where the two functions intersect represents the maximum attainable ${\dot{V}}_{O_{2}}$ and the corresponding $P_{\mathrm{mv}O_{2}}$.

**FIGURE 1:**
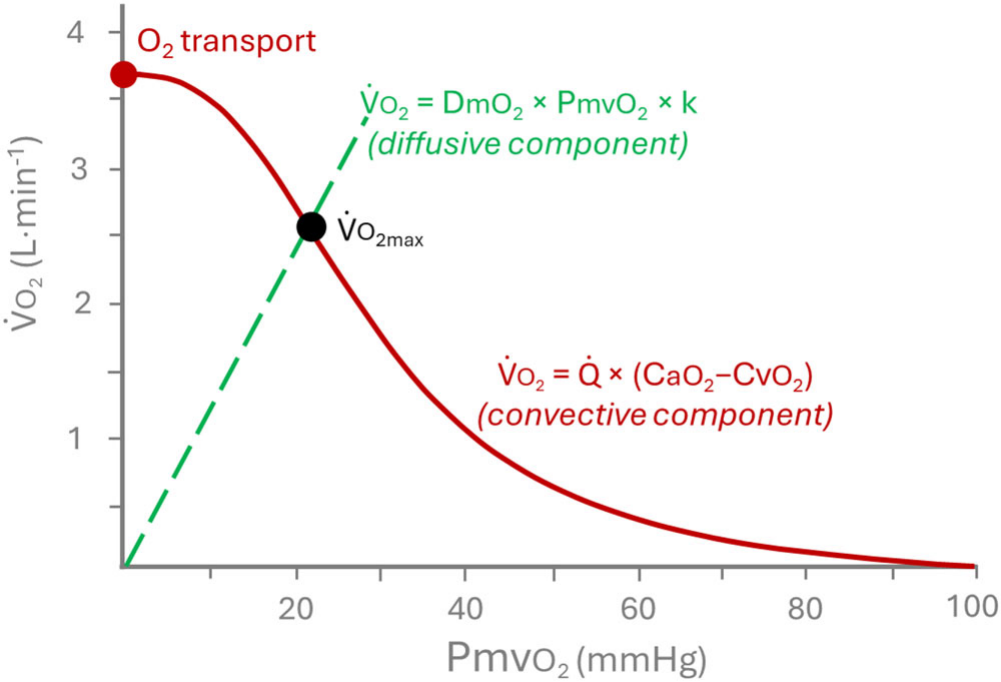
Schematic Wagner diagram representing the convective and muscle diffusive components of oxygen transport. $C_{aO_{2}}$, arterial oxygen concentration; $C_{vO_{2}}$, muscle venous oxygen concentration; $D_{mO_{2}}$, diffusing capacity of muscle for oxygen; $P_{\mathrm{mv}O_{2}}$, muscle venous partial oxygen pressure; $\dot{Q}$, cardiac output; ${\dot{V}}_{O_{2}}$, rate of oxygen uptake; ${\dot{V}}_{O_{2}\max}$, aerobic capacity.

This two‐component model clarifies that ${\dot{V}}_{O_{2}\max}$ is the result of two interacting transport processes rather than a single rate‐limiting step. The dual resistances represented by these two compartments explain why increasing inspired oxygen (hyperoxia) or $\dot{Q}$ in isolation may produce only a small increase in ${\dot{V}}_{O_{2}\max}$ (Wagner, 2006), as sensitivity to such an intervention is determined by the slope of the muscle diffusive component (Wagner, 2023). The two‐component model also provides a mechanistic rationale for why and how much ${\dot{V}}_{O_{2}\max}$ declines with disease (Broxterman et al., 2020; Esposito et al., 2010, 2011; Houstis et al., 2018; Legendre et al., 2021) or deconditioning (Ade et al., 2017) and increases with endurance exercise training (Broxterman et al., 2024; Gifford et al., 2016; Legendre et al., 2021). In these examples, a change in the slope of the muscle diffusive component may ‘sensitize’ the individual to changes in the convective component of oxygen transport. Mass balance dictates that the two transport processes interact to set the maximal value.

Experimental and clinical studies confirm that ${\dot{V}}_{O_{2}\max}$ behaves similarly to the model. Data from studies of hypoxia show that ${\dot{V}}_{O_{2}\max}$ declines at a proportion that can be determined by the slope of the muscle diffusive component (Wagner, 1996a; Wagner et al., 1986). Muscle diseases or chronic obstructive pulmonary disease (COPD) severely impair $D_{mO_{2}}$ resulting in lower ${\dot{V}}_{O_{2}\max}$, and either a similar or potentially greater $P_{\mathrm{mv}O_{2}}$ depending on any compensation or adaptation in the convective component (Broxterman et al., 2020; Wagner, 2023). Alterations in both components can have multiplicative effects. Studies of astronauts after prolonged spaceflight show simultaneous declines in $\dot{Q}$ and $D_{mO_{2}}$ leading to disproportionately large decreases in ${\dot{V}}_{O_{2}\max}$ (Ade et al., 2017). Conversely, highly trained individuals may enhance both components through coordinated adaptations that increase $\dot{Q}$, capillary density and skeletal muscle mitochondrial volume (Roca et al., 1992).

## 3 POTENTIAL CONVECTIVE AND DIFFUSIVE OXYGEN LIMITATIONS IN HTRS

### 3.1 Impaired pulmonary gas exchange

Pulmonary gas exchange impairments, specifically diffusion limitation and alveolar ventilation−perfusion (${\dot{V}}_{A}$/$\dot{Q}$) mismatch, may contribute to constraining exercise capacity in HTRs. Direct evidence supporting pulmonary diffusion impairment in HTRs derives from measurements of lung diffusing capacity for carbon monoxide (*D*_LCO_). Six to twelve months after transplantation, haemoglobin‐adjusted *D*_LCO_ decreased from 83 ± 11% before surgery to 69 ± 12% (*P <* 0.01) (Casan et al., 1987). Similarly, in heart failure patients (stage III and IV), after adjusting for haemoglobin concentration, resting *D*_LCO_ was 73 ± 3% of predicted, and further decreased to 60 ± 2% following heart transplantation (*P <* 0.001; variances are SEM) (Al‐Rawas et al., 2000). After transplantation, *D*_LCO_ was correlated with ${\dot{V}}_{O_{2}\max}$ (*r* = 0.61 to 0.81, *P* ≤ 0. 01) (Ville et al., 1998; Al‐Rawas et al., 2000). However, other reports suggest that only 40−53% of HTRs have impaired lung diffusing capacity (Braith et al., 1993; Ewert et al., 2000; Ville et al., 1998). Of 11 HTRs, only five (45%) had a *D*_LCO_ <70% of predicted, and these individuals experienced exercise‐induced hypoxaemia ($P_{aO_{2}}$ 15–38 mmHg below resting values) (Braith et al., 1993). Limitations of the above studies are that they have small sample size and *D*_LCO_ was determined at rest. Whether *D*_LCO_ changes with exercise in HTRs is unknown and warrants investigation. While the cause of lung diffusion limitation is unknown, it has been speculated that HTRs have irreversible damage of the alveolar capillary membrane from abnormal pulmonary hemodynamics induced by heart failure (Al‐Rawas et al., 2000) and not from cyclosporine (Braith et al., 1993), a calcineurin inhibitor immunosuppressant used to prevent rejection of the cardiac allograft.

Coupled with pulmonary diffusion impairment, HTRs exhibit evidence of ventilatory inefficiency during peak exercise that suggests a ${\dot{V}}_{A}$/$\dot{Q}$ mismatch. Six to twelve months following transplantation, the slope of the ventilatory equivalent for carbon dioxide (${\dot{V}}_{E}/{\dot{V}}_{CO_{2}}$) remained elevated relative to controls (Al‐Rawas et al., 2000). The ${\dot{V}}_{E}/{\dot{V}}_{CO_{2}}$ slope remained above normal (∼32−34) 5 years post‐transplant (Carter et al., 2006). Heart transplant recipients with a low *D*_LCO_ also had an elevated ventilatory equivalent for oxygen (${\dot{V}}_{E}/{\dot{V}}_{O_{2}}$) that was greater than HTRs with normal *D*_LCO_ (∼37 vs. ∼30, respectively, *P <* 0.05) (Ville et al., 1998). However, ventilatory inefficiency may not occur in all HTRs. Eighteen of forty HTRs (46%) had a ${\dot{V}}_{E}/{\dot{V}}_{CO_{2}}$ slope greater than 120% of predicted (Ewert et al., 2000). Another report found that at 2 years post‐transplant, 54% had a ${\dot{V}}_{E}/{\dot{V}}_{CO_{2}}$ slope greater than matched controls (Habedank et al., 2007).

The ventilatory inefficiency in HTRs is consistent with ${\dot{V}}_{A}$/$\dot{Q}$ mismatch, a heightened ventilatory drive and/or diffusion limitation. Yet, additional indirect evidence of a ${\dot{V}}_{A}$/$\dot{Q}$ mismatch comes from observations of a larger physiological dead space fraction of the tidal volume (*V*_D_/*V*_T_) at maximal exercise (0.31 ± 0.02 vs. 0.19 ± 0.01, respectively, *P <* 0.05) and a greater alveolar–arterial oxygen difference ($P_{\left(A-a\right)O_{2}}$) at peak exercise in HTRs than in matched controls (18 ± 2 vs. 14 ± 1 mmHg, respectively; *P <* 0.05; variances are SEM) (Al‐Rawas et al., 2000). Elevated ventilatory equivalents, physiological dead space and $P_{\left(A-a\right)O_{2}}$ are indices of uneven ${\dot{V}}_{A}$/$\dot{Q}$, which causes arterial hypoxaemia, and thus constrains convective oxygen delivery at peak exercise. In addition, elevated *V*_D_/*V*_T_ increases the ventilatory demand for a given ${\dot{V}}_{CO_{2}}$, thereby increasing the work of breathing at any ${\dot{V}}_{O_{2}}$. This has the potential to further constrain convective oxygen delivery to the locomotor muscles at peak exercise through increased activation of the respiratory muscle metaboreflex, posing a competition for the limited $\dot{Q}$, which redirects blood flow away from the locomotor muscles (Harms et al., 1997). In 10 people with heart failure, respiratory muscle unloading at 60% of peak work rate using non‐invasive ventilatory support reduced the work of breathing and increased leg blood flow and leg ${\dot{V}}_{O_{2}}$ by ∼45% (Olson et al., 2010). It is unknown whether locomotor muscle perfusion is also constrained in HTRs by elevated respiratory muscle work consequent to increased *V*_D_/*V*_T_; this mechanism in HTRs deserves further study.

These pulmonary gas exchange abnormalities act synergistically rather than independently: reduced *D*_LCO_ limits alveolar–capillary oxygen equilibration, particularly when mean pulmonary capillary transit time is reduced at maximal exercise; elevated ${\dot{V}}_{E}/{\dot{V}}_{CO_{2}}$ likely reflects increased physiological dead space and results in increased respiratory muscle work for a given level of carbon dioxide clearance; and ${\dot{V}}_{A}$/$\dot{Q}$ mismatch results in failure to fully oxygenate the blood entering the systemic circulation. The net effect of these pulmonary gas exchange abnormalities is a quantitatively meaningful reduction in arterial oxygenation and competition for $\dot{Q}$ that contributes to limiting convective oxygen delivery to the locomotor muscles, at least in ∼40–50% of HTRs. These abnormalities impose a significant upstream limitation on whole‐body oxygen transport.

### 3.2 Impaired oxygen carrying capacity of the blood

Decreased haemoglobin concentration contributes to a diminished convective oxygen transport and is frequently observed in adults (Braith et al., 1993; Müller et al., 2001; Seiler et al., 2021) and children (Newland et al., 2024) following heart transplantation. In heart failure patients with a normal *D*_LCO_, haemoglobin concentration decreased from 14.5 ± 1.7 g dL^−1^ before transplantation to 12.9 ± 1.5 g dL^−1^ at 18 ± 12 months post‐transplant (*P <* 0.05) while those with a low *D*_LCO_ decreased from 14.8 ± 1.4 to 13.1 ± 1.8 g dL^−1^ (*P <* 0.05) (Braith et al., 1993). The decrease in haemoglobin concentration is often severe enough to cause anaemia (Müller et al., 2001; Newland et al., 2024). At 2 years post‐transplant, 18% were still anaemic (haemoglobin < 10.0 g dL^−1^) (Hunt et al., 1992). Through 5 years after transplantation, 43 of 60 HTRs (72%) suffered from mild anaemia (males <14 g dL^−1^, females <12 g dL^−1^) (Müller et al., 2001).

The cause of decreased haemoglobin concentration in HTRs is uncertain, but is associated with the use of CNI (Mitsnefes et al., 2005) and impaired renal function (Gleissner et al., 2004). CNIs are frequently prescribed immunosuppressants to prevent organ rejection following transplantation, and this class of immunosuppressants is well‐recognized to increase the risk of chronic kidney disease (Lee et al., 2007; Ojo et al., 2003). Decreased renal function reduces production of erythropoietin (EPO), a hormone that stimulates haemoglobin production. Indeed, low EPO was reported in anaemic HTRs, which was improved with EPO therapy (Gleissner et al., 2005, 2006).

While the reductions in haemoglobin concentration may be small, it can still have a significant impact on convective oxygen transport. Convective oxygen transport (${\dot{Q}}_{aO_{2}}$) is calculated as the product of $\dot{Q}$ and arterial oxygen concentration,

(4) $${\dot{Q}}_{aO_{2}}=\dot{Q}\times \left[\left(1.34\;\times \mathrm{Hb}\times S_{aO_{2}}\right)+\left(0.003\times P_{aO_{2}}\right)\right]$$

where Hb is haemoglobin concentration, 1.34 mL O_2_ g^−1^ is the empirical average of oxygen that can bind to 1 g of haemoglobin, $S_{aO_{2}}$ is the fractional percentage of oxygen saturation of arterial blood and 0.003 is the solubility coefficient of oxygen in plasma. Assuming $\dot{Q}$ in HTRs is 12 L min^−1^ at peak exercise, $S_{aO_{2}}$ is 96% and the dissolved oxygen in arterial blood is 0.3 mL dL^−1^, then substituting a 2 g dL^−^^1^ reduction in haemoglobin concentration in Equation 4 resolves to a reduction in ${\dot{Q}}_{aO_{2}}$ by ∼300 mL min^−^^1^, or about 14%.

### 3.3 Impaired cardiac output

During recovery from the donor, the cardiac allograft is denervated, which impacts convective oxygen transport in HTRs during vigorous exercise (Kao et al., 1994; Paulus et al., 1992; Scott et al., 2009; Tucker et al., 2018). Resting heart rate (HR) in HTRs is typically 90−110 beats min^−1^ because of the lack of parasympathetic innervation (Kao et al., 1994, 1995; Keteyian et al., 1991; Shah et al., 2013). But during exercise, the lack of sympathetic innervation affects the rise of HR and $\dot{Q}$. Although $\dot{Q}$ at rest and at submaximal exercise is similar to or only mildly reduced compared to age‐matched controls (Marconi & Marzorati, 2003), it was 30–43% lower at peak exercise $\dot{Q}$ (Kao et al., 1994, 1995; Marconi & Marzorati, 2003; Mettauer et al., 1996) largely due to chronotropic and inotropic incompetence and diastolic dysfunction.

To illustrate, in HTRs during the first few minutes of graded exercise, there is little change in HR due to the lack of sympathetic innervation (Figure 2), although augmented venous return and the Frank−Starling mechanism contribute to raising $\dot{Q}$ (Marconi & Marzorati, 2003). After several minutes, rising circulating catecholamines exert chronotropic and inotropic effects on the heart that raise HR and $\dot{Q}$ (Pope et al., 1980). Yet, circulating catecholamines are slow to rise after exercise onset and insufficient to bring about a normal HR response so that submaximal and peak HR and $\dot{Q}$ are considerably lower than in age‐matched healthy individuals. In a review of over 1700 adult HTRs from the literature (mean age = 48 years, range 24 to 60 years) measured more than 1 year after transplantation, the average peak HR was 130 beats min^−1^ (range 110–174 beats min^−1^) (Marconi & Marzorati, 2003).

**FIGURE 2:**
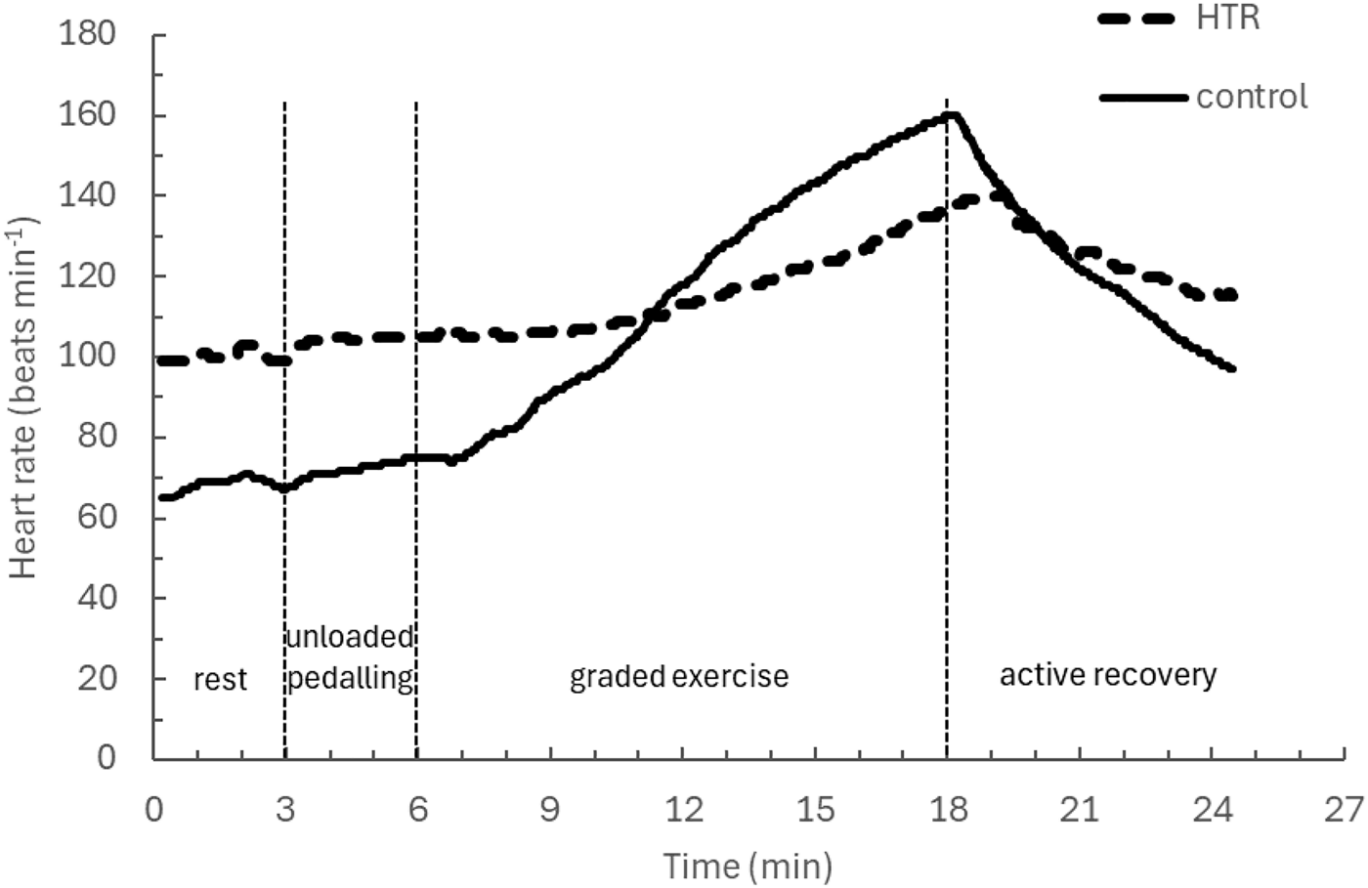
Representation of a HR response to a graded exercise test in a HTR (dashed line) and a healthy age‐matched individual (continuous line) (Sietsema et al., 2020). In the HTR, note the elevated resting HR, sluggish response during the initial period of exercise, diminished peak HR (often occurring 1−2 min after cessation of exercise) and slow HR recovery.

If exercise HR in HTRs contributes to limiting ${\dot{V}}_{O_{2}\max}$, then rate‐responsive HR pacing would be predicted to improve performance. Eight HTRs with rate‐responsive pacemakers (mean age = 57 years) performed two graded exercise tests (Braith et al., 2000). For one test, the pacemakers were turned off so that the exercise HR increased only from circulating catecholamines. On the other test, the pacemakers were programmed to maximal sensitivity, and patients were administered a beta‐blocker to inhibit cardiac effects of circulating catecholamines. When exercise HR was increased only by the pacemaker, the average peak HR and ${\dot{V}}_{O_{2}\max}$ were higher (149 ± 18 vs. 129 ± 16 beats min^−1^; 18.9 ± 5.1 vs. 15.4 ± 4.2 mL kg^−1^ min^−1^) and exercise duration longer (14.6 ± 3.4 vs. 12.4 ± 2.1 min, *P <* 0.05) suggesting that chronotropic incompetence contributes to limiting ${\dot{V}}_{O_{2}\max}$ and exercise tolerance in HTRs (Braith et al., 2000).

Furthermore, any recovery of sympathetic innervation would be expected to increase ${\dot{V}}_{O_{2}\max}$ and exercise performance. After at least 1 year from transplant, 40−55% of HTRs regain some degree of sympathetic reinnervation (Bengel et al., 2001; Buendia‐Fuentes et al., 2011; De Marco et al., 1995; Lovric et al., 2004). Evidence of partial sympathetic reinnervation was observed, primarily in the anteroseptal wall, in 16 of 29 HTRs (Bengel et al., 2001). Heart transplant recipients with reinnervation had a greater peak HR (143 ± 15 vs. 121 ± 13 beats min^−1^, *P <* 0.01) and a longer exercise duration (8.2 ± 1.2 vs. 6.1 ± 1.5 min, *P <* 0.01) on a graded exercise test than those HTRs without reinnervation (Bengel et al., 2001). In a similar finding, peak HR was greater in HTRs who had evidence of partial reinnervation compared to those without (147 ± 15 vs.117 ± 5 beats min^−1^, *P <* 0.005) and had a greater exercise duration (15.1 ± 1.0 vs. 12.4 ± 0.9 min, *P <* 0.01) and ${\dot{V}}_{O_{2}\max}$ (Ciolac et al., 2020). Others suggested that ${\dot{V}}_{O_{2}\max}$ and exercise duration may be greater only in those HTR patients exhibiting marked reinnervation (Wilson et al., 2000).

Denervation of the cardiac allograft is also linked to diastolic dysfunction. In HTR, stroke volume at the point of exercise intolerance is approximately 75% of age‐matched controls (Kao et al., 1994; Scott et al., 2009), a consequence of diastolic dysfunction in the denervated heart (Hausdorf et al., 1989; Rudas et al., 1990). Diastolic dysfunction is correlated with reduced ${\dot{V}}_{O_{2}\mathrm{peak}}$ (Roten et al., 2009). In an intact heart during exercise, cardiac sympathetic stimulation, beyond raising HR, simultaneously activates phospholamban via the cyclic‐AMP pathway. Phospholamban is a cardiac muscle protein that, when phosphorylated, releases its inhibition of cardiac sarcoplasmic reticulum Ca^2+^‐ATPase (SERCA2a) and enhances relaxation and contractility. Consequently, during exercise with a denervated heart, reuptake of sarcoplasmic Ca^2+^ is slower as is relaxation of the ventricles allowing less diastolic filling (Paulus et al., 1992). This effect is expected to be influenced by the degree of reinnervation that could have occurred and by the level of circulating catecholamines. During mild cycling exercise (HR = 100 beats min^−1^), acceleration of left ventricular relaxation from rest was 2.5 times slower in HTRs than a matched control group at the same HR (Paulus et al., 1992). A decrease in ventricular compliance would also be reflected by an elevation of the pulmonary capillary wedge pressure and a smaller end‐diastolic volume. Indeed, HTRs had a 74% greater wedge pressure to end‐diastolic volume index ratio than matched controls at maximal exercise (*P <* 0.001) (Kao et al., 1994). Accordingly, impaired $\dot{Q}$ in HTRs reduces convective oxygen delivery to the locomotor muscles.

### 3.4 Impaired muscle perfusion

Convective oxygen delivery is not only determined by $\dot{Q}$ but is also dependent on the extent to which blood flow is directed to the active musculature. Exercise induces vasodilation of resistance vessels and decreases systemic vascular resistance (SVR), which enhances muscle perfusion and oxygen delivery to the capillary bed of active muscle. While SVR in HTRs is similar to matched controls at rest, it is 34–44% greater at maximal exercise (Kao et al., 1994; Scott et al., 2009) and is moderately correlated with aerobic impairment (*r* = 0.55, *P <* 0.001) (Bussières et al., 1995). Yet, even 12 weeks of exercise training did not improve brachial artery endothelial‐dependent or ‐independent dilation (Haykowsky et al., 2009) or flow‐mediated dilation (Braith et al., 2008) in HTRs despite an increase in ${\dot{V}}_{O_{2}\mathrm{peak}}$.

High SVR appears to be due to at least two factors: increased muscle sympathetic nerve activity (MSNA) and endothelial dysfunction. Heart transplant recipients have greater MSNA (Ciarka et al., 2006; Houssiere et al., 2007) that may result in part from the loss of baroreflex feedback from the denervated heart. Other potential contributing factors include increased peripheral chemoreceptor sensitivity (Ciarka et al., 2006; Houssiere et al., 2007) and muscle metaboreceptor activity (Houssiere et al., 2007). Peripheral endothelial dysfunction, measured from flow‐mediated dilation of the brachial artery, was found in 19 of 40 HTRs (47%), all of whom were prescribed a CNI (Roig et al., 2009). In addition, CNI therapy given to HTRs appears to interfere with exercise‐induced vasodilation. Calcineurin inhibitors reduce nitric oxide (NO) production in cultured rat vascular smooth muscle by decreasing endothelial NO synthase (eNOS), a potent vasodilator (Marumo et al., 1995; Takeda et al., 1999). In human cultured tissue, CNI also stimulate production of endothelin, a potent vasoconstrictor (Miyauchi & Sakai, 2019) (Haug et al., 1995). These simultaneous effects result in shifting of vascular tone toward vasoconstriction. Several investigators observed impaired vasodilation in HTRs during exercise (Bussières et al., 1995; Cuppoletti et al., 2003; Kao et al., 1994; Scott et al., 2009). The diminished ability to vasodilate during exercise negatively affects ${\dot{V}}_{O_{2}\max}$ and exercise performance. The vasodilation response of endothelium‐dependent acetylcholine infusion was moderately correlated (*r* = 0.63) with ${\dot{V}}_{O_{2}\max}$ (*P <* 0.001) (Andreassen et al., 1998). Similarly, flow‐mediated dilation weakly predicted exercise time of HTRs on a graded treadmill test (*r* = 0.34, *P <* 0.001) (Patel et al., 2005).

## 4 EFFECTS OF CALCINEURIN INHIBITORS ON MUSCLE OXYGEN DIFFUSION CAPACITY

Following solid organ transplant, a life‐long regimen of immunosuppressants is necessary to prevent rejection of the donor organ. A combination of different classes of immunosuppressants is used, and the most frequently prescribed class of immunosuppressants is CNIs. Tacrolimus (FK506) and cyclosporin A (CsA) are the most used CNIs, which inhibit T‐cell signal transduction and interleukin 2 (IL‐2) transcription. Calcineurin serves, among other things, as a Ca^2+^‐dependent signalling protein that triggers the immune response (Figure 3). When an antigen binds to a T‐cell receptor, it causes an influx of Ca^2+^ that activates a calcineurin−calmodulin complex. Calcineurin, a phosphatase, then dephosphorylates and activates nuclear factor of activated T‐cells (NFAT), a transcription factor that translocates into the nucleus. Once in the nucleus, the dephosphorylated NFAT upregulates IL‐2, a cytokine that signals the T‐cell immune response.

**FIGURE 3:**
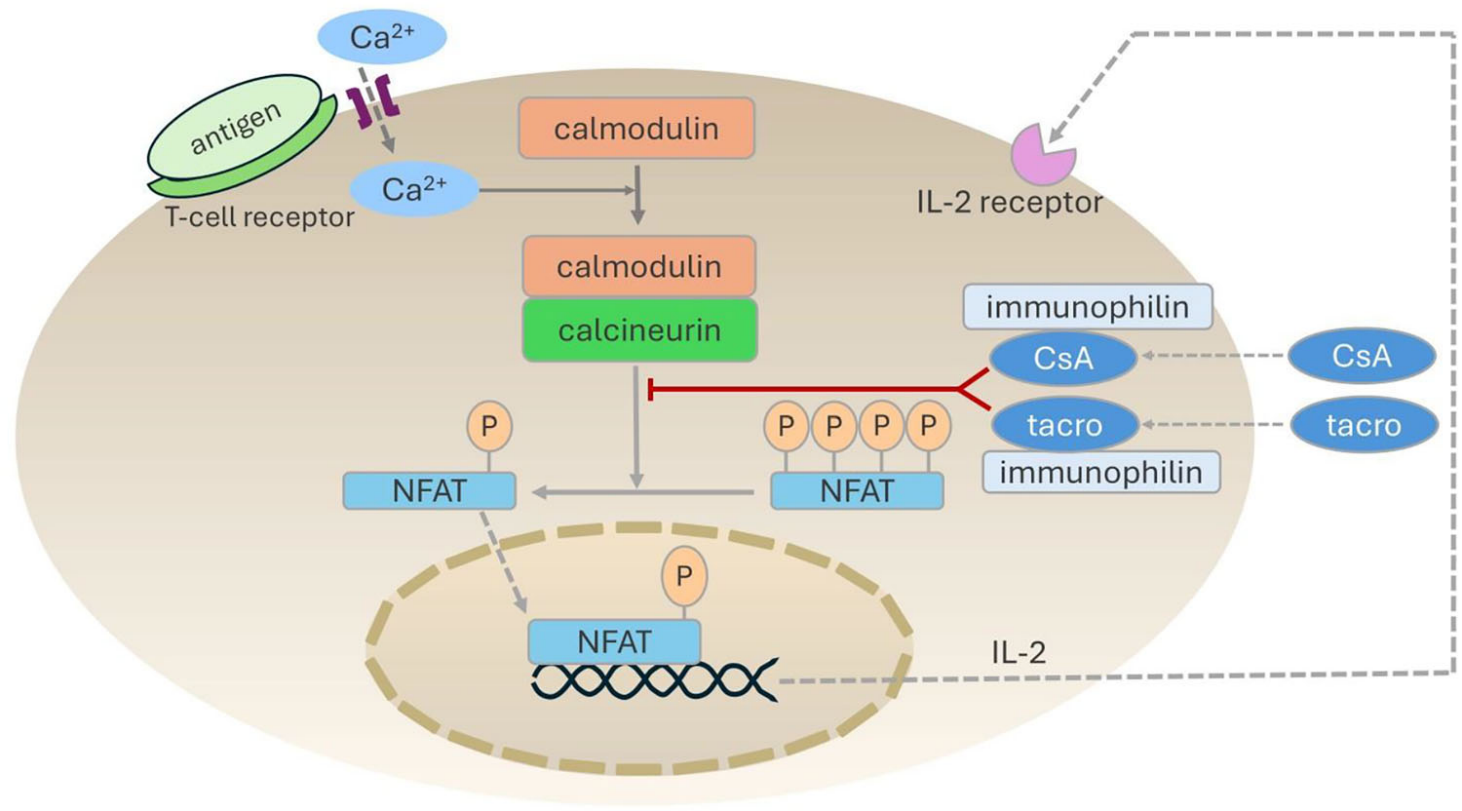
Calcineurin pathway in a T‐cell upregulates interleukin 2 (IL‐2). The CNI tacrolimus (tacro) and cyclosporin A (CsA) inhibit the pathway by preventing dephosphorylation of nuclear factor of activated T‐cells (NFAT).

Calcineurin inhibitors disrupt this pathway by binding to cytoplasmic immunophilins, isomerases, which in turn bind to and inactivate calcineurin, thus preventing it from dephosphorylating NFAT. By inhibiting this pathway, CNIs reduce the immune response and, in solid organ transplants, lessen the risk of organ rejection thereby greatly increasing allograft survival rates (Grattan et al., 1990). As the calcineurin pathway is found in most cells in the body including motor neurons, endothelial cells, and cardiac and skeletal muscle, CNIs have the potential for multiple side effects including ones that could impact exercise tolerance and aerobic capacity. However, the extent to which these effects directly contribute to impaired exercise capacity in HTRs remains incompletely defined.

### 4.1 Effect of CNIs on muscle capillaries

There is evidence of decreased capillary density in skeletal muscle of HTRs that may be related to use of CNIs. In rats, capillary density, capillary‐to‐fibre ratio and the number of capillaries surrounding a fibre were lower in the extensor digitorum longus muscle but not the soleus muscle after 4 weeks of cyclosporine A administration (7 mg kg^−1^ day^−1^) (Biring et al., 1998). Heart failure patients had no change in the number of capillaries surrounding type I or type II fibres by 12 months post‐transplant (Bussières et al., 1997). While the size of type I fibres increased, there was not a concomitant increase in capillary number so that the number of capillaries per fibre area for type I fibres at 12 months post‐transplant was smaller than before transplantation (*P <* 0.05). When compared to a matched control group, heart failure patients had a smaller capillary‐to‐fibre ratio and fewer capillaries in contact with a fibre, which remained lower 6−9 months after transplantation (Schaufelberger et al., 2001). Likewise, HTRs had a 19−24% lower capillary density and 17−27% lower capillary‐to‐fibre ratio compared to matched controls (Lampert et al., 1996, 1998). However, neither capillary measurement correlated with ${\dot{V}}_{O_{2}\mathrm{peak}}$ (Lampert et al., 1996). After 6 weeks of modified interval training, the capillary‐to‐fibre ratio increased 19% in the control group, but not in HTRs, where training failed to induce any capillary growth (Lampert et al., 1998).

One potential mechanism for the decreased skeletal muscle capillarization in HTRs is inhibition of calcineurin–NFAT signalling in endothelial cells by cyclosporin. A major angiogenic growth factor is vascular endothelial growth factor (VEGF). Experimental data indicate that blocking calcineurin–NFAT signalling in endothelial cells can reduce VEGF expression and VEGF receptor‐2 (VEGFR‐2) signalling, thereby blunting VEGF‐driven endothelial proliferation, migration and tube formation (Armesilla et al., 1999; Hernández et al., 2001). Indeed, tacrolimus has been found in mice to inhibit angiogenesis development in vitro and slow breast carcinoma growth in vivo (Siamakpour‐Reihani et al., 2011).

Limitation of the number and proportion of muscle capillaries has a significant effect on ${\dot{V}}_{O_{2}\max}$. This limitation particularly influences the muscle diffusive component of oxygen transport as $D_{mO_{2}}$ depends on the volume and distribution of red blood cells (RBCs) within the muscle capillary network. A reduced capillary supply constrains RBC flux per unit muscle volume, increasing diffusion distances and reducing both the effective surface area for exchange and the capillary–mitochondrion $P_{O_{2}}$ gradient (Malvin & Wood, 1992; Poole & Musch, 2023; Poole et al., 2011). Consequently, oxygen transport from capillary blood to mitochondria is impaired. While this provides a mechanistic basis of CNI in reducing muscle diffusive capacity, direct evidence linking these microvascular alterations to impaired $D_{mO_{2}}$ in HTRs remains speculative.

### 4.2 Effect of CNIs on mitochondrial respiration

Calcineurin inhibitors may affect mitochondrial respiration at high doses, but likely not at therapeutic doses. Several studies reported decreased skeletal muscle mitochondrial respiration in rats following 3 weeks of 20−25 mg kg^−1^ day^−1^ of cyclosporin A treatment (Hokanson et al., 1995; Mercier et al., 1995; Zbreski et al., 2006), doses that are 2.5–5 times therapeutic doses (Lindenfeld et al., 2004). However, the deleterious effect of cyclosporine A on mitochondria may have been due to the high dosage (N'Guessan et al., 2014) and its vehicle (N'Guessan et al., 2014; Sanchez et al., 2000). In a between‐subjects design, compared to a group of pretransplant heart failure patients, HTRs at 15 ± 16 months post‐transplant had a greater muscle oxidative capacity measured from phosphocreatine recovery kinetics using magnetic resonance spectroscopy after maximal arm exercise (Stratton et al., 1994). Since their transplant, HTR patients had been treated with cyclosporine A and azathioprine, and some with prednisone, although whether the immunosuppression treatment was responsible for the greater muscle oxidative capacity post‐transplant could not be determined. In another study, using a repeated‐measure design, oxidative capacity of the vastus lateralis at 10 months post‐transplant was unchanged from before transplant (N'Guessan et al., 2014). Thus, oxidative capacity was unaffected in HTRs who were on a triple‐drug immunosuppressant therapy (cyclosporine A, azathioprine and prednisone). Similarly, observations of normal mitochondrial volume in HTRs were observed at approximately 10 months post‐transplant (Lampert et al., 1996, 1998). This is also consistent with the lack of change in muscle fibre type up to at least 12 months post‐transplant (Bussières et al., 1997; Schaufelberger et al., 2001). While it has been demonstrated in rodents that CNI in high doses for 3 weeks reduced maximal muscle mitochondrial respiration, there exists no evidence that therapeutic doses of CNI in humans leads to reduced mitochondrial volume density, respiratory capacity or maximal rates of oxidative ATP synthesis.

## 5 MODELLING CONVECTIVE AND DIFFUSIVE OXYGEN TRANSPORT DEFICITS IN HTRS

Several recent studies have examined the convective and diffusive oxygen transport mechanisms that limit ${\dot{V}}_{O_{2}\max}$ in chronic heart failure patients (Esposito et al., 2010, 2011; Houstis et al., 2018; Legendre et al., 2021). On average, convective oxygen delivery and muscle oxygen diffusion were 31 ± 0.3% and 36 ± 2% lower, respectively (*P <* 0.001), in heart failure with preserved ejection fraction (HFpEF) patients compared to controls (Houstis et al., 2018). To quantify the effect of each defect, investigators also calculated the theoretical increase in ${\dot{V}}_{O_{2}\max}$ from mathematically ‘correcting’ a single defect to match its corresponding control value. When ${\dot{V}}_{A}$, lung diffusion, and haemoglobin concentration were corrected, ${\dot{V}}_{O_{2}\max}$ was predicted to increase approximately 6%, 6% and 3%, respectively; when $\dot{Q}$ of the HFpEF patients was corrected, ${\dot{V}}_{O_{2}\max}$ was predicted to increase by 7 ± 0.5%; correcting muscle oxygen diffusion capacity predicted an increase in ${\dot{V}}_{O_{2}\max}$ of 27 ± 1% (Houstis et al., 2018).

To generate hypotheses for mechanisms liming ${\dot{V}}_{O_{2}\max}$ in HTRs, we aimed to apply the Wagner convective–diffusive model with exercise data from HTRs. We reviewed the literature for studies on HTRs that measured blood gases and hemodynamic and gas exchange variables at peak exercise. No one study contained all the data necessary to calculate convective oxygen transport and muscle oxygen diffusing capacity, but we identified four HTR studies that included part of the data needed for the calculations. Data for $S_{aO_{2}}$, $P_{aO_{2}}$ and $P_{vO_{2}}$ for both groups and haemoglobin concentration for controls were missing from these studies. The missing values were imputed from four studies using data from heart failure patients for HTRs and corresponding age‐matched controls. Data sources for all the variables are in shown in Table 1. After imputing the missing values, we calculated convective oxygen transport using Equation 4 while muscle oxygen diffusing capacity was determined by Equation 3. Because several missing variables required for these calculations were imputed from non‐heart transplantation studies, the absolute values of ${\dot{Q}}_{aO_{2}}$ and $D_{mO_{2}}$ should be interpreted cautiously. Importantly, inference is driven primarily by the directional pattern (i.e., concurrent reductions in convective oxygen delivery and muscle oxygen diffusing capacity) rather than the exact magnitudes of the estimated deficits. The resulting calculations of ${\dot{Q}}_{aO_{2}}$ and $D_{mO_{2}}$ for HTRs and controls (Table 1) are in line with what others have reported for heart failure patients (Esposito et al., 2010, 2011; Houstis et al., 2018; Legendre et al., 2021), suggesting that the supplemented age‐matched control values used in our retrospective analysis were reasonable assumptions for the missing data. Strength of evidence in Table 1 was assessed qualitatively based on the population specificity (i.e., HTRs), measurement directness, the degree of extrapolation required and a qualitative assessment of the apparent effect size of the difference between HTRs and controls. High evidence indicates direct measurement of key variables in HTRs and age‐matched controls and that further research is unlikely to change confidence in the estimated effect. Moderate evidence means some variables were calculated or drawn from indirect comparisons and that further research may change the estimate of effect. Low evidence indicates that additional research is needed before consistent conclusions can be drawn. Insufficient/imputed evidence indicates that conclusions were extrapolated from non‐HTR populations, imputed values, assumptions or retrospective modelling.

**TABLE 1:** Mean values for calculation of convective and diffusive O_2_ transport at ${\dot{V}}_{O_{2}\max}$ in heart transplant recipients (HTRs) and controls.

	HTRs	Control	∆ %	Strength of evidence
Model inputs				
${\dot{V}}_{O_{2}\max}$ (L min^−1^) (source)	1.14 *n* = 63 (Kao et al., 1994, 1995; Mettauer et al., 1996; Scott et al., 2009)	2.08 *n* = 60 (Kao et al., 1994, 1995; Mettauer et al., 1996; Scott et al., 2009)	−45%	High
$\dot{Q}$ (L min^−1^) (source)	11.2 ± 2.5 *n* = 33 (Kao et al., 1995; Mettauer et al., 1996; Scott et al., 2009)	20.2 ± 4.0 *n* = 25 (Kao et al., 1995; Mettauer et al., 1996; Scott et al., 2009)	−45%	Moderate
∆$a-\bar{v}O_{2}$ (mL O_2_ dL^−1^) (source)	10.9 *n* = 63 (Kao et al., 1994, 1995; Mettauer et al., 1996; Scott et al., 2009)	13.9 *n* = 60 (Kao et al., 1994, 1995; Mettauer et al., 1996; Scott et al., 2009)	−22%	Moderate
Hb (g dL^−1^) (source)	12.9 *n* = 90 (Carter et al., 2006; Kao et al., 1994, 1995)	14.2 *n* = 63 (Esposito et al., 2010; Houstis et al., 2018)	−9%	Low
$S_{aO_{2}}$ (%) (source)	97.8 *n* = 147 (Esposito et al., 2010; Houstis et al., 2018; Legendre et al., 2021)	99.5 *n* = 63 (Esposito et al., 2010; Houstis et al., 2018)	−2%	Insufficient/imputed
$P_{aO_{2}}$ (mmHg) (source)	96 *n* = 91 (Esposito et al., 2010; Houstis et al., 2018)	97 *n* = 63 (Esposito et al., 2010; Houstis et al., 2018)	−1%	Insufficient/imputed
$P_{vO_{2}}$ (mmHg) (source)	24 *n* = 153 (Esposito et al., 2010, 2011; Houstis et al., 2018; Legendre et al., 2021)	21 *n* = 69 (Esposito et al., 2010, 2011; Houstis et al., 2018)	+14%	Insufficient/imputed
**Model outputs**				
${\dot{Q}}_{aO_{2}}$ (L O_2_ min^−1^)	1.9	3.8	−50%	—
$D_{mO_{2}}$ (mL O_2_ min^−1^ mmHg^−1^)	23.7	49.2	−52%	—

*Note*: Values are means ± SD. In cases where SD is not reported, they were not available in the literature.

Abbreviations: ∆$a-\bar{v}O_{2}$, arterial‐mixed venous oxygen difference; Hb, haemoglobin concentration; $P_{aO_{2}}$, arterial partial oxygen pressure; $P_{vO_{2}}$, venous partial oxygen pressure; $\dot{Q}$, cardiac output; ${\dot{Q}}_{aO_{2}}$, convective oxygen transport; $S_{aO_{2}}$, arterial oxygen saturation; ${\dot{V}}_{O_{2}\max}$, maximal pulmonary oxygen uptake.

We applied the results to a Wagner diagram to graphically hypothesize convective and diffusive oxygen transport limitations to ${\dot{V}}_{O_{2}\max}$ in the HTRs and control groups. Figure 4 suggests substantial reductions in convective oxygen delivery and rate of muscle diffusion may exist in HTRs. This hypothesis generating exercise suggests that decreased convective oxygen delivery shifts the Fick principle downward, with impairments of convective oxygen transport influenced by a combination of ${\dot{V}}_{A}$ / $\dot{Q}$ mismatch and/or decreased pulmonary oxygen diffusion, haemoglobin concentration, cardiac output and/or muscle perfusion. The shallower slope of the resultant Fick law of diffusion proposed by these estimations is consistent with reduced skeletal muscle oxygen diffusing capacity, likely reflecting diminished microvascular RBC flux and capillary–mitochondrial oxygen conductance, factors plausibly related to CNI use. Together, the results of these calculations that include direct measurements and imputed data, suggest that reduced convective delivery and reduced diffusing capacity in HTRs shift the intersection – ${\dot{V}}_{O_{2}\max}$ – markedly lower. These estimates highlight that treatment approaches that only address deficits of oxygen delivery in HTRs will be less effective than those that target both convective and diffusive components of oxygen transport. The shallow diffusion slope hypothesized in HTRs means that skeletal muscle is less sensitive to increased convective oxygen delivery.

**FIGURE 4:**
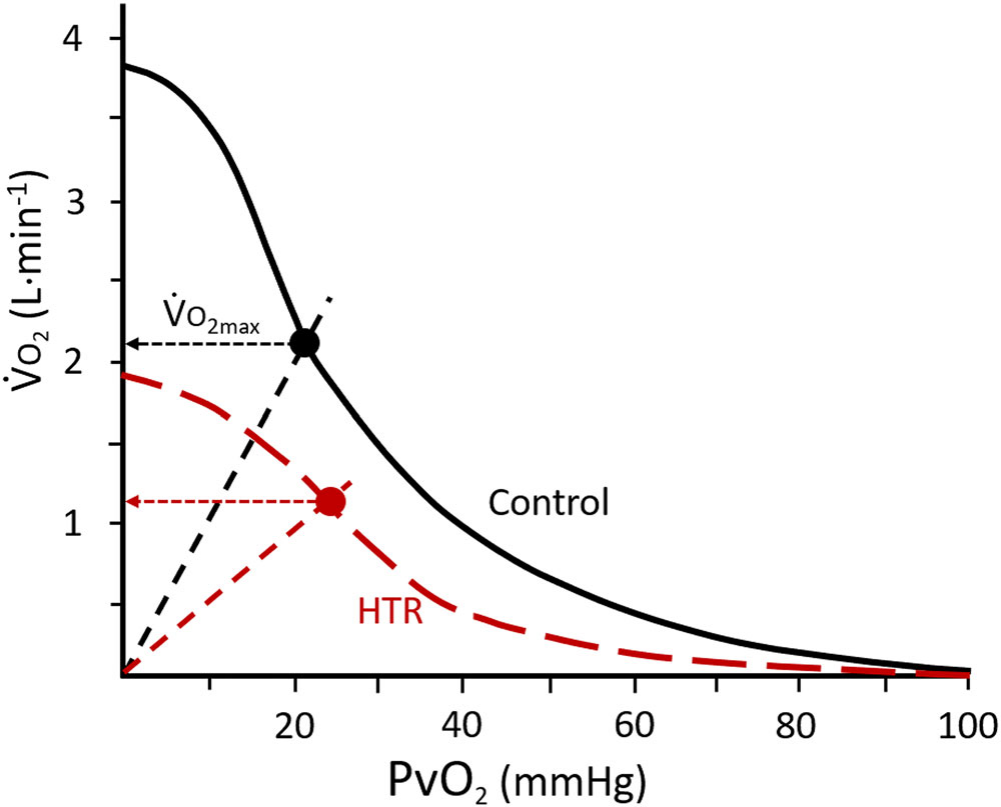
Schematic illustration of convective oxygen transport and muscle oxygen diffusion capacity limitations on ${\dot{V}}_{O_{2}\max}$ between HTRs (long‐dash curve) and matched controls (continuous curve). The lines of muscle oxygen diffusion capacity are indicated by the medium‐dash lines, and the intersection of the convective oxygen transport curve and line of muscle oxygen diffusion capacity is the ${\dot{V}}_{O_{2}\max}$. HTR, heart transplant recipients; $P_{vO_{2}}$, mixed venous partial oxygen pressure; ${\dot{V}}_{O_{2}}$, rate of oxygen uptake; ${\dot{V}}_{O_{2}\max}$, aerobic capacity.

As the steps of oxygen transport are serially linked, there is an interaction between components. Each component can affect ${\dot{V}}_{O_{2}\max}$ (Wagner, 2023) with varying sensitivity. Deficits at multiple steps compound rather than substitute for one another (Houstis et al., 2018). For example, a modest impairment in $C_{aO_{2}}$ combined with a modest reduction in peak $\dot{Q}$ produces a larger decrement in systemic oxygen delivery and ${\dot{V}}_{O_{2}\max}$ than either limitation alone. Importantly, excess capacity at one step cannot compensate for a deficit at another (Wagner, 2023): a high mitochondrial oxidative capacity cannot overcome inadequate oxygen delivery, nor can an elevated $\dot{Q}$ fully offset reduced $C_{aO_{2}}$ or impaired muscle diffusing capacity. Within Wagner's convective–diffusive model, this serial dependence is reflected by the intersection of the convective delivery curve and the muscle diffusion line. When both shift unfavourably, the resulting reduction in ${\dot{V}}_{O_{2}\max}$ is disproportionately large, underscoring why multiple modest abnormalities can produce severe exercise intolerance.

Because several parameters used in the Wagner diagram analysis were obtained from non‐heart transplant populations, a sensitivity analysis was performed to assess the robustness of the derived estimates of convective oxygen transport and muscle oxygen diffusing capacity. Each borrowed parameter was varied individually across a physiologically plausible range (i.e., $S_{aO_{2}}$ ± 1%, $P_{aO_{2}}$ ± 10 mmHg, $P_{vO_{2}}$ ± 4 mmHg), while all other variables were held constant. The resulting changes in ${\dot{Q}}_{aO_{2}}$ and $D_{mO_{2}}$ were quantified as percent change (Table 2).

**TABLE 2:** Sensitivity analysis of varying $S_{aO_{2}}$, $P_{aO_{2}}$ and $P_{vO_{2}}$ on estimates of convective and diffusive oxygen transport in HTRs.

Variable	Range tested	Δ${\dot{Q}}_{aO_{2}}$	∆$D_{mO_{2}}$
$S_{aO_{2}}$	96−99%	0% to −1%	0%
$P_{aO_{2}}$	85−105 mmHg	Negligible	0%
$P_{vO_{2}}$	20−28 mmHg	0%	−14% to −20%

*Note*: $D_{mO_{2}}$, diffusing capacity of muscle for oxygen; $P_{aO_{2}}$, arterial partial oxygen pressure; $P_{vO_{2}}$, the venous partial oxygen pressure; ${\dot{Q}}_{aO_{2}}$, convective oxygen transport; $S_{aO_{2}}$, the fractional percentage of oxygen saturation of arterial blood.

The sensitivity analysis demonstrated that variation in arterial oxygenation ($S_{aO_{2}}$, $P_{aO_{2}}$) had minimal influence on calculated convective oxygen transport, reflecting the small contribution of dissolved oxygen to arterial oxygen content. In contrast, $P_{vO_{2}}$ exerted a greater influence on estimates of muscle oxygen diffusing capacity. However, even when $P_{vO_{2}}$ was varied across physiologically plausible ranges, the calculated reduction in $D_{mO_{2}}$ in HTRs remained substantial. These findings indicate that the observed impairment in diffusive oxygen transport in HTRs is likely robust and not attributable to uncertainty of the imputed data. Furthermore, the estimates for ${\dot{V}}_{O_{2}\max}$ and peak $\dot{Q}$ are supported by direct measurements from HTR patients and therefore represent the strongest evidence in the model. In contrast, estimates involving $P_{vO_{2}}$ and $D_{mO_{2}}$ should be interpreted more cautiously because they depend on non‐HTR data.

Several limitations in this retrospective analysis warrant consideration. Our assessments of the convective and diffusive oxygen transport considered the entire periphery rather than an individual muscle, a practice that has been used by others (Ade et al., 2017; Houstis et al., 2018; Legendre et al., 2021; Simonson et al., 2015). Thus, muscle blood flow, oxygen uptake and diffusive capacity of the exercising muscles would be lower than the whole‐body values we report. Despite systematic errors of the absolute values for the exercising muscles, the relative comparisons remain robust. In addition, data from studies differing in immunosuppressive and adjunctive medication regimens introduce potential variability that cannot be fully accounted for. Although CNIs provide a plausible mechanistic link to peripheral limitations, the present analysis cannot isolate CNI‐specific effects from other contributors to impaired muscle oxygen transport and utilization in HTRs. We did not include an analysis of how much physical‐inactivity‐driven deconditioning contributes to the hypothesized reductions in convective and diffusive oxygen transport because we were not able to source data to quantify this. However, the finding that exercise training does not consistently reverse some of the deficits in HTRs suggest that deconditioning is not the sole contributor to observed impairments in aerobic capacity. While we recognize the inherent limitations of our analysis, we suggest multiple factors are affecting convective and diffusive oxygen transport to limit ${\dot{V}}_{O_{2}\max}$ in HTRs. Our retrospective analysis is intended to be hypothesis‐generating, and given the data available, these limitations of ${\dot{V}}_{O_{2}\max}$ appear both reasonable and worthy of further investigation.

## 6 SUMMARY

Endurance exercise tolerance is mediated by transport and utilization of oxygen, as demonstrated by a high ${\dot{V}}_{O_{2}\max}$. In both health and disease, ${\dot{V}}_{O_{2}\max}$ depends on multiple variables that contribute to convective oxygen transport, muscle oxygen diffusing capacity and muscle mitochondrial oxidative capacity. Heart transplant recipients have a low whole‐body aerobic capacity, but they differ from heart failure patients in that they have a denervated heart, and most are taking CNIs for immunosuppression, both of which may negatively influence key components of oxygen transport. The lack of sympathetic control of the heart during exercise results in a diminished heart rate response and smaller stroke volume causing a lower peak $\dot{Q}$. Calcineurin inhibitors may contribute to reduced muscle perfusion and capillary density. In addition, convective oxygen transport in HTRs may be diminished by decreased pulmonary gas exchange efficiency related to impaired diffusion capacity, ${\dot{V}}_{A}$/$\dot{Q}$ mismatch and a decreased haemoglobin concentration. While the definitive experiment to measure convective and diffusive limitations in HTRs has yet to be performed, values to estimate these variables were drawn from a range of literature, which support that deficits in both oxygen convection and muscle diffusion constrain ${\dot{V}}_{O_{2}\max}$. This analysis rationalizes the need for direct, prospective measurement of these components in HTRs. Given the growing number of HTRs and the strong association between exercise capacity, longevity and quality of life, direct assessment of convective and diffusive components at peak exercise represents an important research priority to target specific interventional treatments. While the Wagner model can quantify the relative contributions of convective delivery and muscle diffusion limitation, it does not by itself identify the upstream causes of each defect. Notably, the peripheral effects of CNIs on vascular and skeletal muscle physiology remain under‐studied in this context.

## AUTHOR CONTRIBUTIONS

Fred W. Kolkhorst conceived and drafted manuscript; Fred W. Kolkhorst prepared figures and tables; Fred W. Kolkhorst, Daniel T. Cannon, Harry B. Rossiter edited and revised the final version of manuscript. All authors have read and approved the final version of this manuscript and agree to be accountable for all aspects of the work in ensuring that questions related to the accuracy or integrity of any part of the work are appropriately investigated and resolved. All persons designated as authors qualify for authorship, and all those who qualify for authorship are listed.

## CONFLICT OF INTEREST

Fred W. Kolkhorst and Daniel T. Cannon declare no conflicts of interest, financial or otherwise. Harry B. Rossiter reports consulting fees from the NIH RECOVER‐ENERGIZE working group (1OT2HL156812) and is involved in contracted clinical research with Biocient, Intervene Immune, Mezzion, Regeneron, Respira and Roche. He is a visiting Professor at the University of Leeds, UK and the University of Pavia, Italy. He is a member of the Scientific Advisory Board for MGC Diagnostics. He reports US/EU patent applications filed by The Lundquist Institute, titled ‘Testing System to Diagnose Neuromuscular Deconditioning and Pathologic Conditions’. He receives royalties as a contributor to the textbook, *Wasserman and Whipp's Principles of Exercise Testing and Interpretation*.

## GENERATIVE AI STATEMENT

During preparation of this manuscript, ChatGPT was used for editorial assistance, including improving clarity, organization, and wording of selected sections. All scientific content, interpretations, and references were independently verified by the authors, who take full responsibility for the final manuscript.
